# QualitySNP: a pipeline for detecting single nucleotide polymorphisms and insertions/deletions in EST data from diploid and polyploid species

**DOI:** 10.1186/1471-2105-7-438

**Published:** 2006-10-09

**Authors:** Jifeng Tang, Ben Vosman, Roeland E Voorrips, C Gerard van der Linden, Jack AM Leunissen

**Affiliations:** 1 Plant Research International, PO Box 16, 6700 AA Wageningen, The Netherlands; 2 Laboratory of Bioinformatics, Wageningen University, PO Box 8128, 6700 ET Wageningen, The Netherlands

## Abstract

**Background:**

Single nucleotide polymorphisms (SNPs) are important tools in studying complex genetic traits and genome evolution. Computational strategies for SNP discovery make use of the large number of sequences present in public databases (in most cases as expressed sequence tags (ESTs)) and are considered to be faster and more cost-effective than experimental procedures. A major challenge in computational SNP discovery is distinguishing allelic variation from sequence variation between paralogous sequences, in addition to recognizing sequencing errors. For the majority of the public EST sequences, trace or quality files are lacking which makes detection of reliable SNPs even more difficult because it has to rely on sequence comparisons only.

**Results:**

We have developed a new algorithm to detect reliable SNPs and insertions/deletions (indels) in EST data, both with and without quality files. Implemented in a pipeline called QualitySNP, it uses three filters for the identification of reliable SNPs. Filter 1 screens for all potential SNPs and identifies variation between or within genotypes. Filter 2 is the core filter that uses a haplotype-based strategy to detect reliable SNPs. Clusters with potential paralogs as well as false SNPs caused by sequencing errors are identified. Filter 3 screens SNPs by calculating a confidence score, based upon sequence redundancy and quality. Non-synonymous SNPs are subsequently identified by detecting open reading frames of consensus sequences (contigs) with SNPs. The pipeline includes a data storage and retrieval system for haplotypes, SNPs and alignments. QualitySNP's versatility is demonstrated by the identification of SNPs in EST datasets from potato, chicken and humans.

**Conclusion:**

QualitySNP is an efficient tool for SNP detection, storage and retrieval in diploid as well as polyploid species. It is available for running on Linux or UNIX systems. The program, test data, and user manual are available at  and as Additional files.

## Background

Sequence variation in the genomic DNA of individuals of the same species or related species are typically single nucleotide polymorphisms (SNP) or small insertions/deletions (indels) [1,2]. Because of their abundance and slow mutation rate within the genome, they are the most common type of genetic markers [3] for studying complex genetic traits and genome evolution [4]. In addition SNPs in coding sequences can be used to directly study the genetics of expressed genes and to map functional traits [5,6]. Non-synonymous SNPs (nsSNPs) are of particular interest because they change the protein sequence, possibly affecting protein function [7,8].

There are several strategies, both experimental and computational for SNP discovery. Experimental SNP discovery often consists of a number of laborious steps that make this process complex and expensive [2]. The computational approach makes use of the large sequence datasets present in public databases. Over the last few years, a number of pipelines have been developed that automatically detect SNPs in such databases. One type of pipeline detects SNPs using trace files or quality files, for example the PHRED/PHRAP/PolyBayes system [9,10] and other pipelines [2,3,11-14]. The other type of pipeline uses only EST redundancy in text-based sequence files to detect SNPs: these include autoSNP [15,16] and SNiPpER [17]. Both autoSNP and SNiPpER are based on sequence redundancy for the initial detection of SNPs, and sequencing errors are detected and filtered out by analyzing SNP patterns.

The major drawback of all these computational approaches is that they do not provide a good way to distinguish allelic variation from sequence variation between paralogous sequences. In addition, they do not recognize sequencing errors very well, leading to the frequent occurrence of false positives [7,16,18]. Only PolyBayes [9] has implemented an enhanced paralog identification routine, but it requires the corresponding genomic sequence and quality files in addition to the EST sequence. As most public ESTs do not include trace or quality file, and genomic sequences are not available for most species, the versatility of the PolyBayes paralog identification routine is limited.

In this paper we describe a new method (QualitySNP) that uses a haplotype-based strategy to detect reliable synonymous and non-synonymous SNPs from public EST data without the requirement of trace/quality files or genomic sequence data. Haplotypes in this context represent the different alleles of a gene in a dataset. The haplotype reconstruction is based on a mathematical algorithm. QualitySNP's versatility is demonstrated by the identification of SNPs in EST datasets from potato, chicken and humans.

## Materials

For potato, two datasets were used in our study. One dataset was from the EMBL database (version 79), containing 83,565 ESTs with tissue information of the potato variety Kennebec. The other was from the Potato Gene Index of TIGR database (data of Dec 7th 2004) containing 87,637 reads of potato ESTs with quality files, which was used to evaluate the quality of public potato ESTs and the performance of our SNP discovery pipeline. Function annotation information of the potato ESTs was obtained from the TIGR Gene Index [19] and UniGene [20]. For chicken, a dataset consisting of 100,000 ESTs, originating from more than one genotype was used [21]. For thorough validation of our program, nineteen UniGene datasets obtained from NCBI (Build #191 of *Homo sapiens*) were used. The Single Nucleotide Polymorphism database (dbSNP) was downloaded from NCBI (Jun 3th 2006) to our local machine.

To detect non-synonymous and synonymous SNPs, the UniProt database (version of Feb 28th 2005) [22] was used to obtain reference protein sequences for ORF detection; FASTY, which is a module of the FASTA package 3.4 [23] and BLAST package 2.2.10 [24] were used as tools for searching the UniProt database. CAP3 [25] was used for assembling sequences. Cross_match [26] was used for removing vector fragments; the vector sequences were downloaded from the NCBI data repository on Dec 5th 2004. To verify paralog identification the BLAT server of the human genome reference sequence was used [27].

## Architectural structure

**Figure 1:**
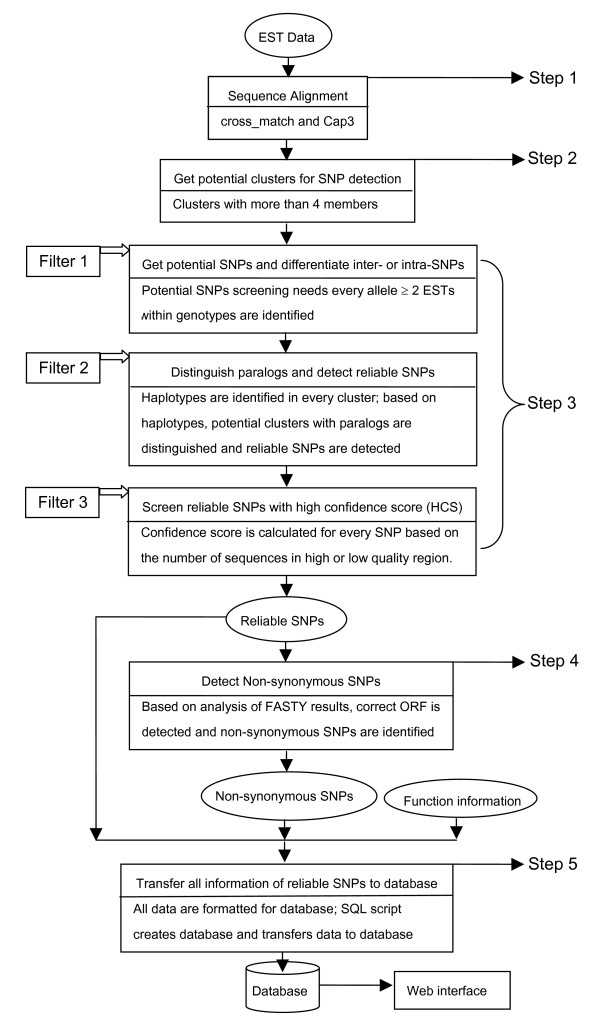
Flowchart for detecting reliable SNPs in the QualitySNP pipeline. Step1 through 5 are described in detail in paragraph "Architectural structure"

The pipeline consists of five steps: 1) EST assembling using cross_match for removing vectors and CAP3 for sequence clustering, 2) analysis of the alignment information to select clusters with at least 4 EST members, 3) SNP detection and distinguishing variations between or within genotypes, 4) distinction between non-synonymous and synonymous SNPs using FASTY, and 5) transferring the final results into a SNP database (Figure 1). The pipeline is implemented in standard C-Shell script on a Linux workstation; the individual programming steps are written in the C programming language, with exception of the alignment analysis tool (PERL5.8) and the web pages to view the results from the database (PHP4 and MYSQL3.23).

In step 3 three filters are used to detect reliable SNPs: filter 1 screens clusters for potential SNPs and differentiates variations between or within genotypes; filter 2 detects clusters containing variations caused by sequencing errors and paralogous sequences; and filter 3 detects unreliable SNPs by assigning confidence scores to SNPs based on sequence redundancy and sequence quality.

## Implementation

### Filter 1: screening for potential SNPs

EST data are clustered by CAP3 with a stringency level of 95% similarity per 100 bp, which is also used by other SNP mining programs [12,15]; this setting is sufficient to prevent clustering of paralogous sequences in most cases. Clusters with at least 4 members are extracted from the alignment information, as well as annotation information, which was obtained from the TIGR Gene Index or UniGene [20]. We detect all potential SNPs including bi-, tri-, and tetra-allelic SNPs, with the requirement that every allele is represented by more than one sequence [16,28] (see Figure 1; filter 1). If genotype information for sequences is available, it can be used to classify SNPs as occurring between and/or within genotypes.

### Filter 2: screening for reliable SNPs

#### (1) Haplotype reconstruction

In our setup, a haplotype is defined as a group of sequences within a cluster that represent the same allele of a gene. All the sequences in a haplotype should therefore have the same nucleotide on every polymorphic site. Our program reconstructs haplotypes using a mathematical method that minimizes false haplotype reconstruction due to the occurrence of sequencing errors (see below).

Firstly, the similarity *S*_*ij *_per polymorphic site between candidate sequence *i *and all current members of one potential haplotype is defined as

Sij=∑k=1msij(k)∑k=1msij(k)+∑k=1mdij(k)     [1]
 MathType@MTEF@5@5@+=feaafiart1ev1aaatCvAUfKttLearuWrP9MDH5MBPbIqV92AaeXatLxBI9gBaebbnrfifHhDYfgasaacH8akY=wiFfYdH8Gipec8Eeeu0xXdbba9frFj0=OqFfea0dXdd9vqai=hGuQ8kuc9pgc9s8qqaq=dirpe0xb9q8qiLsFr0=vr0=vr0dc8meaabaqaciaacaGaaeqabaqabeGadaaakeaacqWGtbWudaWgaaWcbaGaemyAaKMaemOAaOgabeaakiabg2da9maalaaabaWaaabmaeaacqWGZbWCdaWgaaWcbaGaemyAaKMaemOAaOgabeaakiabcIcaOiabdUgaRjabcMcaPaWcbaGaem4AaSMaeyypa0JaeGymaedabaGaemyBa0ganiabggHiLdaakeaadaaeWaqaaiabdohaZnaaBaaaleaacqWGPbqAcqWGQbGAaeqaaOGaeiikaGIaem4AaSMaeiykaKcaleaacqWGRbWAcqGH9aqpcqaIXaqmaeaacqWGTbqBa0GaeyyeIuoakiabgUcaRmaaqadabaGaemizaq2aaSbaaSqaaiabdMgaPjabdQgaQbqabaGccqGGOaakcqWGRbWAcqGGPaqkaSqaaiabdUgaRjabg2da9iabigdaXaqaaiabd2gaTbqdcqGHris5aaaakiaaxMaacaWLjaWaamWaaeaacqaIXaqmaiaawUfacaGLDbaaaaa@615F@

where *j *is one polymorphic site of the sequence *i*, *k *is one current member of the potential haplotype, and *m *is the total number of current members in the potential haplotype. *s*_*ij*_(*k*) expresses whether or not the nucleotide at polymorphic site *j *of sequence *i *is the same as that of member *k *in the haplotype, whereas *d*_*ij*_(*k*) expresses whether it is different: when the nucleotide at site *j *in sequence *i *is the same as that in sequence *k*, *s*_*ij*_(*k*) is set to 1 and *d*_*ij*_(*k*) is set to 0; when the nucleotides are different, *s*_*ij*_(*k*) is set to 0 and *d*_*ij*_(*k*) is set to 1. If sequence *k *has no information at site *j *both *s*_*ij*_(*k*) and *d*_*ij*_(*k*) are set to 0. *S*_*ij *_is the similarity of sequence *i *to all current members in the potential haplotype on site *j*; *D*_*ij *_is the dissimilarity between them. When *S*_*ij *_is more than 0.75, sequence *i *is considered to match the haplotype on site *j*, so *S*_*ij *_is set to 1 and *D*_*ij *_is set to 0. When *S*_*ij *_as calculated from [1] is less than 0.75 there is not enough information to assign sequence *i *to the potential haplotype with confidence, so *S*_*ij *_is set to 0 and *D*_*ij *_is set to 1. When both ∑k=1msij(k)
 MathType@MTEF@5@5@+=feaafiart1ev1aaatCvAUfKttLearuWrP9MDH5MBPbIqV92AaeXatLxBI9gBaebbnrfifHhDYfgasaacH8akY=wiFfYdH8Gipec8Eeeu0xXdbba9frFj0=OqFfea0dXdd9vqai=hGuQ8kuc9pgc9s8qqaq=dirpe0xb9q8qiLsFr0=vr0=vr0dc8meaabaqaciaacaGaaeqabaqabeGadaaakeaadaaeWaqaaiabdohaZnaaBaaaleaacqWGPbqAcqWGQbGAaeqaaaqaaiabdUgaRjabg2da9iabigdaXaqaaiabd2gaTbqdcqGHris5aOGaeiikaGIaem4AaSMaeiykaKcaaa@3AC9@ and ∑k=1mdij(k)
 MathType@MTEF@5@5@+=feaafiart1ev1aaatCvAUfKttLearuWrP9MDH5MBPbIqV92AaeXatLxBI9gBaebbnrfifHhDYfgasaacH8akY=wiFfYdH8Gipec8Eeeu0xXdbba9frFj0=OqFfea0dXdd9vqai=hGuQ8kuc9pgc9s8qqaq=dirpe0xb9q8qiLsFr0=vr0=vr0dc8meaabaqaciaacaGaaeqabaqabeGadaaakeaadaaeWaqaaiabdsgaKnaaBaaaleaacqWGPbqAcqWGQbGAaeqaaaqaaiabdUgaRjabg2da9iabigdaXaqaaiabd2gaTbqdcqGHris5aOGaeiikaGIaem4AaSMaeiykaKcaaa@3AAB@ are 0, *S*_*ij *_and *D*_*ij *_are set to 0.

Secondly, the similarity *S*_*i *_of sequence *i *and the potential haplotype of all polymorphic sites is defined as

Si=∑j=1nSij∑j=1nSij+∑j=1nDij     [2]
 MathType@MTEF@5@5@+=feaafiart1ev1aaatCvAUfKttLearuWrP9MDH5MBPbIqV92AaeXatLxBI9gBaebbnrfifHhDYfgasaacH8akY=wiFfYdH8Gipec8Eeeu0xXdbba9frFj0=OqFfea0dXdd9vqai=hGuQ8kuc9pgc9s8qqaq=dirpe0xb9q8qiLsFr0=vr0=vr0dc8meaabaqaciaacaGaaeqabaqabeGadaaakeaacqWGtbWudaWgaaWcbaGaemyAaKgabeaakiabg2da9maalaaabaWaaabmaeaacqWGtbWudaWgaaWcbaGaemyAaKMaemOAaOgabeaaaeaacqWGQbGAcqGH9aqpcqaIXaqmaeaacqWGUbGBa0GaeyyeIuoaaOqaamaaqadabaGaem4uam1aaSbaaSqaaiabdMgaPjabdQgaQbqabaaabaGaemOAaOMaeyypa0JaeGymaedabaGaemOBa4ganiabggHiLdGccqGHRaWkdaaeWaqaaiabdseaenaaBaaaleaacqWGPbqAcqWGQbGAaeqaaaqaaiabdQgaQjabg2da9iabigdaXaqaaiabd6gaUbqdcqGHris5aaaakiaaxMaacaWLjaWaamWaaeaacqaIYaGmaiaawUfacaGLDbaaaaa@55D2@

where *n *is the total number of all potential polymorphic sites of sequence *i*. When S_*i *_is more than 0.8, sequence *i *is considered to belong to this haplotype. If both ∑j=1nSij
 MathType@MTEF@5@5@+=feaafiart1ev1aaatCvAUfKttLearuWrP9MDH5MBPbIqV92AaeXatLxBI9gBaebbnrfifHhDYfgasaacH8akY=wiFfYdH8Gipec8Eeeu0xXdbba9frFj0=OqFfea0dXdd9vqai=hGuQ8kuc9pgc9s8qqaq=dirpe0xb9q8qiLsFr0=vr0=vr0dc8meaabaqaciaacaGaaeqabaqabeGadaaakeaadaaeWaqaaiabdofatnaaBaaaleaacqWGPbqAcqWGQbGAaeqaaaqaaiabdQgaQjabg2da9iabigdaXaqaaiabd6gaUbqdcqGHris5aaaa@376E@ and ∑j=1nDij
 MathType@MTEF@5@5@+=feaafiart1ev1aaatCvAUfKttLearuWrP9MDH5MBPbIqV92AaeXatLxBI9gBaebbnrfifHhDYfgasaacH8akY=wiFfYdH8Gipec8Eeeu0xXdbba9frFj0=OqFfea0dXdd9vqai=hGuQ8kuc9pgc9s8qqaq=dirpe0xb9q8qiLsFr0=vr0=vr0dc8meaabaqaciaacaGaaeqabaqabeGadaaakeaadaaeWaqaaiabdseaenaaBaaaleaacqWGPbqAcqWGQbGAaeqaaaqaaiabdQgaQjabg2da9iabigdaXaqaaiabd6gaUbqdcqGHris5aaaa@3750@ are 0, the value of *S*_*i *_is set to 0.0.

#### (2) Identification of paralogs

Sets containing paralogous sequences can be expected to contain more polymorphisms than sets with only allelic sequences. A method based on the number and frequency of polymorphisms may therefore separate paralogs from alleles. However, some EST clusters show a larger than average number of SNPs because some genes or regions of genes evolve more rapidly [29]. These SNPs represent allelic variation but will be mistaken for variation between paralogs by such an approach. Therefore we developed a method that identifies paralogs by using the differences in SNP numbers between potential haplotypes of the same cluster. The standard deviation of the number of potential SNPs among potential haplotypes in one cluster is calculated and used to identify haplotypes likely to be caused by paralogous sequences. The procedure of identifying paralogs is as follows:

(a) Remove all potential haplotypes consisting of only one sequence: these are probably of poor quality [30].

(b) Calculate the number of potential SNPs defining each haplotype, i.e. the number of potential SNP sites where all sequences in all other haplotypes contain the same nucleotide and only the current haplotype has a different nucleotide.

(c) Normalize this number of SNPs defining each potential haplotype:

nrm_snpi=snpi∑i=1ahapsnpiahap{i|i∈[1,ahap]},
 MathType@MTEF@5@5@+=feaafiart1ev1aaatCvAUfKttLearuWrP9MDH5MBPbIqV92AaeXatLxBI9gBaebbnrfifHhDYfgasaacH8akY=wiFfYdH8Gipec8Eeeu0xXdbba9frFj0=OqFfea0dXdd9vqai=hGuQ8kuc9pgc9s8qqaq=dirpe0xb9q8qiLsFr0=vr0=vr0dc8meaabaqaciaacaGaaeqabaqabeGadaaakeaafaqabeqacaaabaGaemOBa4MaemOCaiNaemyBa0Maei4xa8Laem4CamNaemOBa4MaemiCaa3aaSbaaSqaaiabdMgaPbqabaGccqGH9aqpdaWcaaqaaiabdohaZjabd6gaUjabdchaWnaaBaaaleaacqWGPbqAaeqaaaGcbaWaaSaaaeaadaaeWaqaaiabdohaZjabd6gaUjabdchaWnaaBaaaleaacqWGPbqAaeqaaaqaaiabdMgaPjabg2da9iabigdaXaqaaiabdggaHjabdIgaOjabdggaHjabdchaWbqdcqGHris5aaGcbaGaemyyaeMaemiAaGMaemyyaeMaemiCaahaaaaaaeaadaGadaqaaiabdMgaPjabcYha8jabdMgaPjabgIGiopaadmaabaGaeGymaeJaeiilaWIaemyyaeMaemiAaGMaemyyaeMaemiCaahacaGLBbGaayzxaaaacaGL7bGaayzFaaaaaiabcYcaSaaa@66A1@

where *snp*_*i *_(*i *∈ [1, *ahap*]) is the number of potential SNPs defining haplotype *i*, and *ahap *is the number of all haplotypes after removing poor quality haplotypes (a).

(d) Calculate the standard deviation of the normalized number of potential SNPs among these haplotypes:

D=∑i=1ahap(nrm_snpi−1)2ahap     [3]
 MathType@MTEF@5@5@+=feaafiart1ev1aaatCvAUfKttLearuWrP9MDH5MBPbIqV92AaeXatLxBI9gBaebbnrfifHhDYfgasaacH8akY=wiFfYdH8Gipec8Eeeu0xXdbba9frFj0=OqFfea0dXdd9vqai=hGuQ8kuc9pgc9s8qqaq=dirpe0xb9q8qiLsFr0=vr0=vr0dc8meaabaqaciaacaGaaeqabaqabeGadaaakeaacqWGebarcqGH9aqpdaGcaaqaamaalaaabaWaaabmaeaadaqadaqaaiabd6gaUjabdkhaYjabd2gaTjabc+faFjabdohaZjabd6gaUjabdchaWnaaBaaaleaacqWGPbqAaeqaaOGaeyOeI0IaeGymaedacaGLOaGaayzkaaWaaWbaaSqabeaacqaIYaGmaaaabaGaemyAaKMaeyypa0JaeGymaedabaGaemyyaeMaemiAaGMaemyyaeMaemiCaahaniabggHiLdaakeaacqWGHbqycqWGObaAcqWGHbqycqWGWbaCaaaaleqaaOGaaCzcaiaaxMaadaWadaqaaiabiodaZaGaay5waiaaw2faaaaa@52F2@

In theory, the value of D can range from 0 to infinite. In our study, in 98% of the clusters the value of D ranged from 0 to 1. With increasing D-value the variation in number of SNPs among haplotypes is larger, and there is a higher probability of paralogs in the cluster. The value of D can therefore be used to identify clusters with a low probability of containing paralogs. Following from its definition D-value can only be used to distinguish paralogous clusters if at least three haplotypes are identified in those clusters.

#### (3) Identification of reliable SNPs

In addition to using a redundancy-based criterion (all potential SNPs need at least 2 ESTs for every allele), another more stringent selection is used in the algorithm. The selection is a combination of two measures: major allele haplotype score and minor allele haplotype score. The major allele is the allele occurring in the majority of the sequences in a cluster, while the other is called the minor allele. The major allele haplotype score (*mahap*) is defined as the number of haplotypes with a major allelic nucleotide on one polymorphic site, and the minor allele haplotype score (*mihap*) is the number of haplotypes with the minor allelic nucleotide. The formulas are as follows:

mahap=∑i=1ahapmahapi{mahapi=1|wh×hai+wl×laihci≥0.75}     [4]
 MathType@MTEF@5@5@+=feaafiart1ev1aaatCvAUfKttLearuWrP9MDH5MBPbIqV92AaeXatLxBI9gBaebbnrfifHhDYfgasaacH8akY=wiFfYdH8Gipec8Eeeu0xXdbba9frFj0=OqFfea0dXdd9vqai=hGuQ8kuc9pgc9s8qqaq=dirpe0xb9q8qiLsFr0=vr0=vr0dc8meaabaqaciaacaGaaeqabaqabeGadaaakeaacqWGTbqBcqWGHbqycqWGObaAcqWGHbqycqWGWbaCcqGH9aqpdaaeWaqaaiabd2gaTjabdggaHjabdIgaOjabdggaHjabdchaWnaaBaaaleaacqWGPbqAaeqaaaqaaiabdMgaPjabg2da9iabigdaXaqaaiabdggaHjabdIgaOjabdggaHjabdchaWbqdcqGHris5aOWaaiWaaeaacqWGTbqBcqWGHbqycqWGObaAcqWGHbqycqWGWbaCdaWgaaWcbaGaemyAaKgabeaakiabg2da9iabigdaXiabcYha8naalaaabaGaem4DaCNaemiAaGMaey41aqRaemiAaGMaemyyae2aaSbaaSqaaiabdMgaPbqabaGccqGHRaWkcqWG3bWDcqWGSbaBcqGHxdaTcqWGSbaBcqWGHbqydaWgaaWcbaGaemyAaKgabeaaaOqaaiabdIgaOjabdogaJnaaBaaaleaacqWGPbqAaeqaaaaakiabgwMiZkabicdaWiabc6caUiabiEda3iabiwda1aGaay5Eaiaaw2haaiaaxMaacaWLjaWaamWaaeaacqaI0aanaiaawUfacaGLDbaaaaa@7677@

mihap=∑i=1ahapmihapi{mihapi=1|wh×hbi+wl×lbihci≥0.75}     [5]
 MathType@MTEF@5@5@+=feaafiart1ev1aaatCvAUfKttLearuWrP9MDH5MBPbIqV92AaeXatLxBI9gBaebbnrfifHhDYfgasaacH8akY=wiFfYdH8Gipec8Eeeu0xXdbba9frFj0=OqFfea0dXdd9vqai=hGuQ8kuc9pgc9s8qqaq=dirpe0xb9q8qiLsFr0=vr0=vr0dc8meaabaqaciaacaGaaeqabaqabeGadaaakeaacqWGTbqBcqWGPbqAcqWGObaAcqWGHbqycqWGWbaCcqGH9aqpdaaeWaqaaiabd2gaTjabdMgaPjabdIgaOjabdggaHjabdchaWnaaBaaaleaacqWGPbqAaeqaaaqaaiabdMgaPjabg2da9iabigdaXaqaaiabdggaHjabdIgaOjabdggaHjabdchaWbqdcqGHris5aOWaaiWaaeaacqWGTbqBcqWGPbqAcqWGObaAcqWGHbqycqWGWbaCdaWgaaWcbaGaemyAaKgabeaakiabg2da9iabigdaXiabcYha8naalaaabaGaem4DaCNaemiAaGMaey41aqRaemiAaGMaemOyai2aaSbaaSqaaiabdMgaPbqabaGccqGHRaWkcqWG3bWDcqWGSbaBcqGHxdaTcqWGSbaBcqWGIbGydaWgaaWcbaGaemyAaKgabeaaaOqaaiabdIgaOjabdogaJnaaBaaaleaacqWGPbqAaeqaaaaakiabgwMiZkabicdaWiabc6caUiabiEda3iabiwda1aGaay5Eaiaaw2haaiaaxMaacaWLjaWaamWaaeaacqaI1aqnaiaawUfacaGLDbaaaaa@76AD@

*ha*_*i *_and *la*_*i *_are the number of sequences with the major allelic nucleotide occurring in high quality and low quality regions (this will be described in detail in the next section, filter 3); *hb*_*i *_and *lb*_*i *_are the number of sequences with the minor allelic nucleotide represented in high quality and low quality regions; *wh *and *wl *are the weight values for the high quality and low quality regions; *hc*_*i *_is the number of sequences in the haplotype *i *with information at the polymorphic site. When more than 75% of the members in one haplotype have the same major or minor allelic nucleotide, *mahap*_*i *_or *mihap*_*i *_is increased by 1; otherwise they remain the same, as in this case the correct nucleotide on this site of the haplotype can not be assigned easily. Note that in "Haplotype reconstruction" in filter 2, we allowed for some discrepancies between haplotype members. When both *mahap *and *mihap *are greater than 1 in the cluster, each of major and minor allele occurs in at least one haplotype and the SNP therefore can be considered to be reliable.

SNP patterns and SNP blocks are also defined in filter 2. SNP patterns are those SNPs with the same pattern of allele distribution over the haplotypes; they are determined as in the autoSNP program [16]. SNP blocks are defined as sets of adjacent SNPs with the same SNP pattern. SNP pattern and SNP block information is part of the output of the pipeline, and can be used for instance for linkage disequilibrium (LD) studies.

### Filter 3: screening SNPs with high confidence score (HCS)

The third filter calculates a confidence score for every putative SNP according to the number of occurrences of each allele in high (HQ) and low (LQ) quality regions. In standard sequencing procedures the beginnings and the ends of the sequence are generally of lower quality than the rest of the sequence, and therefore are likely to contain more sequencing errors. We used potato ESTs with quality scores from the TIGR database to establish the boundaries of HQ and LQ for sequences (see Results), and these were used as default settings in our program.

**Figure 2:**
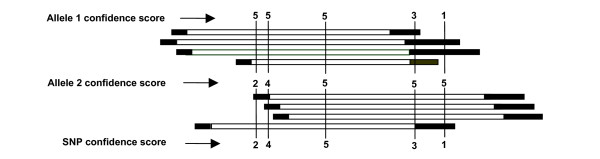
Scoring rules for SNP confidence scores. The SNP confidence score is the smaller one of bi-allele confidence scores. Rectangles in black and white represent low quality regions (LQ) and high quality region (HQ) respectively. The confidence scores for each allele are as follows: 5 if the allele occurs in more than one HQ; 4 if in one HQ and at least two LQ; 3 if in more than 3 LQ; 2 if in one HQ and one LQ, or in 3 LQ; 1 if in 2 LQ, otherwise 0.

Based on the HQ and LQ, the confidence score of each allele is calculated according to the score rules as defined in figure 2. The SNP confidence score is the smaller one of each allele confidence score. The confidence scores for each allele are as follows: 5 if the allele occurs in more than one HQ; 4 if in one HQ and at least two LQ; 3 if in more than 3 LQ; 2 if in one HQ and one LQ, or in 3 LQ; 1 if in 2 LQ, otherwise 0 (Figure 2). In our study, we assigned a high confidence score (HCS) to SNPs with a confidence score of at least 2. This threshold can be adjusted by users according to their specific requirements.

If quality files of sequences are available, one additional filtering step is used to screen for reliable SNPs. In this filter "SNP quality" is calculated as the smaller value of the average quality scores of major and minor alleles per polymorphic site in a cluster. In our study we used a minimum (PHRED) score value of 20.

### Non-synonymous SNP identification

For the detection of non-synonymous SNPs (nsSNP), synonymous SNPs and SNP in UTR, two strategies can be used: alignment with reference protein sequences or ORF prediction using programs such as ESTscan. In our approach, the first method is used; FASTY was chosen as the tool to search the protein database rather than BLASTX, because it allows for frameshifts within codons [31] and produces better alignments with poor sequences. In our potato EST analysis the UniProt database was chosen as referencing database. After the step 3 of our SNP detection program the FASTY results are used by a parsing program, together with the alignment information and the SNP information, to identify the SNP type (nsSNP, sSNP or SNP in 3' UTR or 5' UTR). For this, the FASTY result is first sorted by E-value to get the hit with highest similarity. Next, any frameshift in the contig is detected and corrected, after which the ORF is detected. Finally, all nsSNPs and sSNPs in the protein hit region or coding region, and SNPs or indels in 3' UTR or 5' UTR are identified.

### Database and SNP information retrieval system

All files containing relevant SNP information are transferred to the database. An SQL script for SNP database creation and data loading is produced automatically by the pipeline. The data in this database can be made accessible through the use of a web server. PHP scripts for generating web pages are supplied together with the code of the pipeline. The PHP script allows for easy retrieval of SNP information from the database, and BLAST searching (for an example, see our website [32]).

## Results

The new pipeline for SNP detection (called QualitySNP) presented here distinguishes itself from other programs mainly in the approach it takes for detecting sequencing errors and paralogous sequences. The source code and the manual of the program are freely available for academic use [[32], see Additional file 1, 3], an sample dataset for testing QualitySNP is available as well [[32], see Additional file 2]. To demonstrate the specific properties and advantages of our program we have used potato, human and chicken ESTs as a target for SNP identification. Potato was chosen because it is a tetraploid species and cultivars consist of clonally propagated, heterozygous genotypes. The high level of heterozygosity and the tetraploid nature present problems for most currently available SNP detection programs in particular in the discrimination of paralogs from alleles. Also, within the genomes of plants large numbers of duplications are found [33,34] which may complicate detection of reliable SNPs. Human and chicken datasets were used as a reference for a 'normal' diploid species and to illustrate specific properties and advantages of QualitySNP.

### Predicting haplotypes

In the first step, 83,565 potato ESTs were assembled into 10,670 clusters (Figure 1, step 1), of which 4864 clusters contained 4–100 members (Figure 1, step 2). After the analysis of alignment information of these 4864 clusters, 3081 clusters with potential SNPs were detected (Figure 1, step 3, filter 1). These 3081 clusters contained 41,532 ESTs (average of 14 ESTs per cluster) and 31,815 potential SNPs (average of 10 SNPs per cluster). In the tetraploid potato a maximum of 4 haplotypes per plant can be expected. For most haplotypes sufficient redundancy was available to use a similarity threshold per SNP site (*S*_*ij*_, formula [1]) of 75%: for haplotypes with less than 4 members, all sequences must have the same nucleotide at a single SNP site, whereas for haplotypes with 4 or more members at least 75% of the sequences must match the consensus nucleotide.

In the current dataset a contig was on average 1200 nucleotides in length, and ESTs were about 500~600 nucleotides long, so every EST would contain about 5 potential SNP sites on average. The frequency of sequence errors for EST data from NCBI dbEST was found to be 1 in 500 nucleotides [35]. Assuming a similar error rate, on average one of the five potential SNPs per EST observed in our data would be a sequencing error. With this in mind, the similarity threshold (*S*_*i*_, formula [2]) for the whole sequence was set to 80% for every EST in one haplotype. With these settings, eighty-five percent of the clusters contained at most four haplotypes (Table 1); when haplotypes with only one member were excluded the percentage of clusters with at most four haplotypes increased to 96% (Table 1), which agrees with the tetraploid nature of the potato cultivar Kennebec.

**Table 1:** Relationship of the number of haplotypes and the number of clusters derived from potato variety "Kennebec".

No. of haplotypes in one cluster	No. of clusters^*a*^	No. of clusters with ahap^*b*^
2	1478	1924
3	679	680
4	452	347
5	253	99
6	131	24
7	51	5
8	25	0
more than 8	12	2
total clusters with SNPs	3081	3081
clusters with at most 4 haplotypes	2609(84.7%)	2951(95.8%)

^*a *^all haplotypes in clusters were counted; ^*b *^when clusters contained at least 2 haplotypes with at least 2 members, only these haplotypes were counted.

The thresholds of 75% and 80% are the default values in our program, but they can be adjusted by users according to their specific requirements.

### Predicting paralogs based on haplotypes

**Figure 3:**
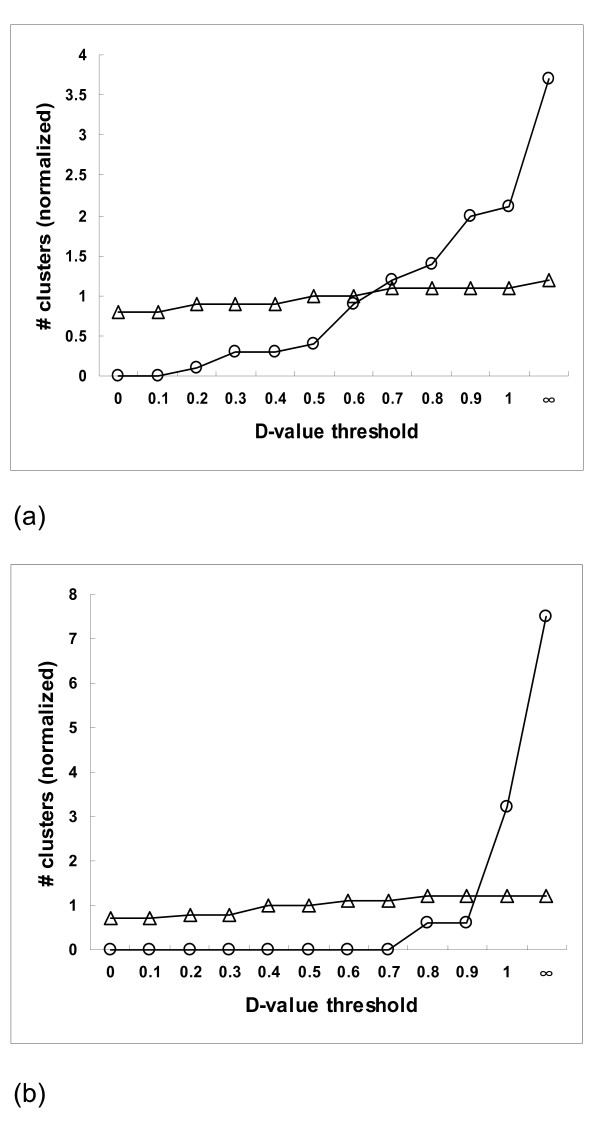
The relationship of the normalized number of clusters in the datasets containing allelic sequences and paralogs. The dataset contained allelic sequence(clusters with 4–20 members; △) and those contained paralogs (clusters with 100–300 members; ○) with the threshold for D-value using (a) the potato data, and (b) the chicken data.

Our method uses the standard deviation (D) of the (normalized) number of SNPs per haplotype to identify clusters that probably contain paralogs (See formula [3]). In order to get a D-value threshold, we assumed that clusters with 4–20 members contained mostly allelic sequences [10], and all clusters with at least 100 members paralogous sequences [10,16]. Under this assumption, 2,544 clusters from the potato dataset were considered to be allelic clusters and 28 clusters with 100 to 300 members paralogous clusters. Figure 3a shows the relationship of the D-value threshold with the paralogous and allelic data set, after normalizing these data. With increasing D-value threshold, the number of presumably paralogous clusters increased sharply. The number of allelic clusters hardly changed. Both lines were found to be crossing at a D-value threshold of approximately 0.6, which was considered optimal for the screening of paralogs in the potato dataset. From the presumed paralogous dataset 17.8 % (5 clusters) of the clusters had D-values less than 0.6 and were most likely not paralogous clusters but for instance allelic clusters of highly expressed single genes (called false negative), and 9.8% (252 clusters) from the presumed allelic sequence dataset with D-value more than 0.6 may be clusters with sequences from lowly expressed paralogous genes (called false positive) (Figure 3a). Using this default setting, 2651 (86%) clusters had a D-value lower than 0.6, and these were therefore considered to be most likely free of paralogs. We used the same approach to determine the D-value threshold a chicken EST dataset of 100,000 sequences. There were 3,426 clusters with between 4–20 members and 23 clusters with 100–300 members used to get the D-value threshold. In this case lines were found to be crossing at D-value 0.9 (Figure 3b), and 8.7% (2) of the clusters from the presumed paralogous dataset may in fact be allelic clusters from highly expressed genes (false negatives), whereas 4.5% (153) of the clusters of the allelic set most likely contained lowly expressed paralogs (false positives).

### Evaluation of paralogs identification

To evaluate the paralog identification routine of QualitySNP, 15 human UniGene datasets from NCBI and the human genomic sequence from UCSC were analyzed. QualitySNP was executed on these UniGene datasets individually after clustering by CAP3. The clusters with a D-value higher than 0.6 (default setting) were considered as clusters containing paralogs. For most clusters identified by QualitySNP as paralogous clusters, the consensus sequences (as generated by CAP3) were compared to the human genomic sequence by the BLAT server of UCSC. For 49 of the 62 (79%) presumed paralogous clusters the consensus sequences picked up multiple loci in the genome (with similarity setting 90% for 90% of the whole sequence) (see Table 2). Further analysis of some of these clusters revealed that separate paralogous genes on the human genome were indeed represented in the clusters. The majority of the clusters identified as paralogous clusters by QualitySNP were from UniGene datasets which were related to gene families. For instance, Unigene Hs.510635 located on 14q32.33 and in this region at least 9 genes (IGHD, IGHG1, IGH@, IGHG3, IGHA2, IGHM, IGHG2, IGHA1, IGHG4) are present that are highly similar. For this Unigene dataset different haplotypes in a paralogous cluster were also found to correspond to different genes (IGHA1 and IGHD1).

**Table 2:** Paralog identification by QualitySNP in human UniGene datasets.

	D-value > = 0.6			D-value > = 0.9		
	
UniGene	No. of cluster^*a*^	confirmed	unconfirmed	No. of cluster^*b*^	confirmed	unconfirmed
Hs.300701	4	4	0	0	0	0
Hs.533717	4	0	4	1	0	1
Hs.12956	3	0	3	1	0	1
Hs.22543	1	1	0	1	1	0
Hs.468478	1	1	0	1	1	0
Hs.591503	1	0	1	0	0	0
Hs.567284	1	0	1	0	0	0
Hs.510172	1	0	1	1	0	1
Hs.406754	10	10	0	4	4	0
Hs.510635	29	28	1	16	16	0
Hs.61635	1	1	0	0	0	0
Hs.631881	1	0	1	0	0	0
Hs.104741	1	0	1	0	0	0
Hs.534639	1	1	0	0	0	0
Hs.18069	3	3	0	1	1	0

Total	62	49(79.03%)	13(20.97%)	26	23 (88.46%)	3 (11.54%)

^*a *^clusters with D value more than 0.6 are considered as clusters containing paralogs by QualitySNP; ^*b *^clusters with D value more than 0.9 are considered as clusters containing paralogs. Confirmed: Number of clusters that were proven to contain paralogous sequences

When the D-value was increased to 0.9, the number of clusters identified as potentially paralogous decreased from 62 to 26; 88% of those clusters (23) were verified by the BLAT analysis against the human genome.

### Predicting reliable SNPs

**Figure 4:**
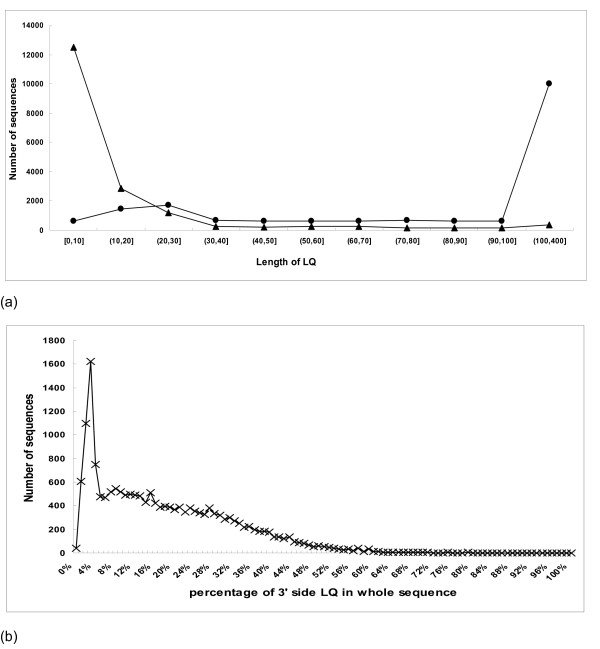
The distribution of low quality region of TIGR potato sequences. (a) Frequency distribution of the size of the low quality region (LQ). The size of LQ from the 5' side is the line with black triangles; the size of LQ from the 3' side is the line with black circles. (b) As 4a, with the size of the 3' LQ region expressed as percentage of the sequence length.

The quality of the SNP data was further improved by taking the quality of the sequence data into account. This was demonstrated with the potato EST dataset, Figure 4 shows the relationships between the length of the low quality region and the number of potato EST sequences. The threshold for high quality region of sequences was a minimum average PHRED score of 20 in a 50 nucleotides sliding window. From figure 4 it is clear that the low quality region (LQ) at the beginning (5' end) of sequences was shorter than the LQ region at the 3' end. At the 5' side of the sequences, 90% had a LQ of less than 30 nucleotides (Figure 4a). At the 3'-end, a large number of sequences had LQs of over 100 nt. Setting a fixed nucleotide limit would either exclude many sequences with short LQs, or include many sequences with large LQs at the 3'-side. Figure 4b shows that there is a relationship between the length of the LQ at the 3'-side and the total length of the sequence. Therefore we set the LQ to 30 nucleotides from the 5' side and 20% of the whole sequence for the 3' side as the default settings. In formula [4] and [5] for *mahap *and *mihap*, the default value of the weight values for HQ (*wh*) or LQ (*wl*) were set to 1.0, but these can be adjusted according to the data quality. For example, if sequences in low quality regions are very bad, the parameters can be set to *wl *= 0.5 and *wh *= 1.0. In filter 3 confidence scores are calculated (see Implentation section, filter 3).

Using the default settings the major and minor allele haplotype score were calculated and this resulted in a selection of 17,745 reliable SNPs from the potato EST dataset from a total of 31,815. An additional 1,020 SNPs with confidence scores less than 2 were dropped from the set of SNPs which left 16,725 reliable bi-SNPs including 1815 indels. The ratio of transitions (C for T or A for G) and transversion (C for G/A, G for C/T, A for C/G) was 1.9 (9853/5057), the frequency of reliable SNPs was one SNP per 224 nucleotides, and the frequency of indels was one per 2,070 nucleotides (further statistic information is presented at our website [32]).

**Figure 5:**
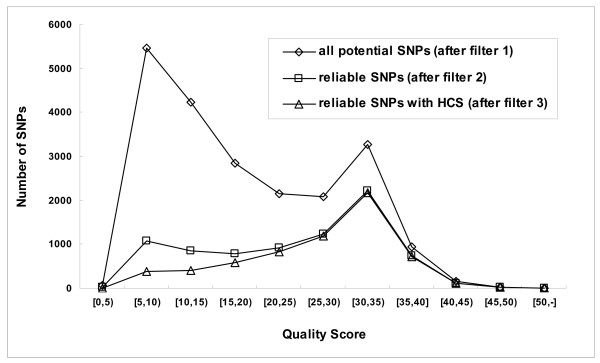
The distribution of potential (filter 1), reliable (filter 2) and HCS (filter 3) SNPs over the PHRED quality scores. High confidence score (HCS) means the SNP confidence score is at least 2. The EST dataset used for this analysis included quality files obtained from the TIGR potato gene index.

The accuracy of the determination of SNP reliability from sequence data only was evaluated by using another potato EST dataset from TIGR that included quality files. In this dataset 21,240 potential SNPs were detected by filter 1, of these 7,971 reliable SNPs were identified by filter 2, and 6,431 were attributed a high confidence score, (HCS, confidence score at least 2) by filter 3. The SNP quality score was calculated by an additional filter from quality files (see Implementation section, filter 3). The distribution of potential (filter 1), reliable (filter 2) and HCS (filter 3) SNPs over the PHRED quality scores is shown in figure 5. After filter 2, 66% of the reliable SNPs had a quality score above 20. Applying filter 3 increased the percentage of reliable HCS SNPs with a quality score above 20 to 78%.

### Predicting non-synonymous SNPs

Depending on the research context, users of the pipeline may have an interest in predicting non-synonymous SNPs. This is in particular the case for studies involving protein structure and functional domains, where SNPs might affect the function of a protein. The UniProt database was searched with the consensus sequences of 2651 clusters of potato dataset (selected with a D-value threshold of 0.6). FASTY identified 2167 (81.7%) contigs with an open reading frame (ORF) that matched entries in the UniProt database, including 102 contigs which were corrected for frameshifts. This indicates that the UniProt had sufficient coverage to act as reference protein database for potato. Using the FASTY results, 10,354 reliable SNPs were identified in protein-encoding regions, 34% of these being nsSNP, which is similar to the results obtained by other authors: 35% in chicken ESTs [8], 32% in Arabidopsis [36].

### Validation of reliable SNPs and comparison with other programs

Reliable SNPs as identified by QualitySNP were validated by experimental data for the potato dataset. For this, we used 37 amplified and sequenced loci containing 60 SNPs and one indel identified by QualitySNP in the potato variety Kennebec (Van der Linden et al, in prep.). Three SNPs turned out to be false, and for 8 SNPs the resequencing data was not conclusive, most likely due to the tetraploid nature of potato. The remaining 50 SNPs as well as the indel were confirmed, demonstrating the reliability of the SNPs identified by Quality SNP.

Up to now, PolyBayes is considered to be one of the best SNP detection programs. However, the program needs the availability of the EST trace files or quality files or the genomic sequence in order to perform its task. This limits the usability of PolyBayes and similar programs, such as PolyFreq [14] to cases for which these conditions are met, and therefore excludes the use of EST datasets for which no genomic sequences or quality files are available yet. autoSNP does not suffer from these limitations. We therefore compared the performance of QualitySNP with AutoSNP on an identical dataset.

QualitySNP and AutoSNP were used to identify SNPs in nineteen individual human UniGene datasets. For each cluster of EST sequences, the consensus sequence was used to BLAST against SNP loci of dbSNP (default settings E-value is 0.01). Each SNP from dbSNP that occurs in the consensus sequence was determined by finding the perfect match of its sequence context in the consensus sequence. An SNP is considered to be confirmed when the SNP locus (approx. 60nt) matches the consensus sequence for 90% or more and the SNP identity (location and substitution) is confirmed. The results are summarized in Table 3. As dbSNP is not complete (only part of the potential SNPs in the human genome are represented in the database), a number of SNPs as identified in the EST dataset will not match dbSNP entries. Nevertheless, in total 35% of the SNPs identified by QualitySNP were confirmed. This was over four times more than for SNPs identified by autoSNP (8%). QualitySNP identified most of the confirmed SNPs found by AutoSNP. Moreover, most of the confirmed SNPs detected by autoSNP were also detected by QualitySNP. In addition, QualitySNP (a C-program) calculated SNP much more efficient than autoSNP (a Perl program); it used less CPU time for calculation than autoSNP, which is especially evident when large clusters are present.

**Table 3:** Validation of SNPs detected by QualitySNP and autoSNP in nineteen UniGene data sets of human.

			QualitySNP(D < = 0.6)				autoSNP				their overlap		
			
chromosome	UniGene	Size	Time(m)	candidate SNPs^*a*^	confirmed	unconfirmed	Time(m)	candidate SNPs^*b*^	confirmed	unconfirmed	candidate SNPs	confirmed	unconfirmed
6	Hs.300701	3640	2	18	5 (27.8%)	13	150	6	0 (0%)	6	3	0(0%)	3
7	Hs.401316	1090	1	0	0 (0%)	0	3	4	0 (0%)	4	0	0(0%)	0
14	Hs.533717	1601	1	12	3 (25%)	9	26	166	1 (0.6%)	165	0	0(0%)	0
17	Hs.12956	622	1	10	2 (20%)	8	1	15	1 (6.7%)	14	9	1(11.11%)	8
19	Hs.515126	654	1	1	0 (0%)	1	2	44	0 (0%)	44	1	0(0%)	1
15	Hs.22543	847	1	10	1 (10%)	9	1	4	1 (25%)	3	1	1(100%)	0
2	Hs.468478	183	1	0	0 (0%)	0	1	0	0 (0%)	0	0	0(0%)	0
1	Hs.591503	200	1	6	2 (33.3%)	4	1	5	0 (0%)	5	3	0(0%)	3
6	Hs.567284	194	1	7	0 (0%)	7	1	8	0 (0%)	8	7	0(0%)	7
6	Hs.510172	282	1	1	0 (0%)	1	1	0	0 (0%)	0	0	0(0%)	0
17	Hs.406754	6453	2	49	25 (51%)	24	51	43	6 (14%))	37	14	5(35.71%)	9
14	Hs.510635	27193	4	535	198 (37%)	337	13	895	92 (10.3%)	803	143	86(60.14%)	57
7	Hs.61635	82	1	0	0 (0%)	0	1	0	0 (0%)	0	0	0(0%)	0
2	Hs.631881	355	1	5	0 (0%)	5	1	1	0 (0%)	1	0	0(0%)	0
8	Hs.104741	275	1	0	0 (0%)	0	1	0	0 (0%)	0	0	0(0%)	0
2	Hs.534639	1910	1	11	1 (9.1%)	10	6	9	0 (0%)	9	6	0(0%)	6
14	Hs.18069	1965	1	3	1 (33.3%)	2	1	1	0 (0%)	1	0	0(0%)	0
17	Hs.514220	6800	2	8	2 (25%)	6	267	13	0 (0%)	13	2	0(0%)	2
12	Hs.19192	397	1	1	0 (0%)	1	2	0	0 (0%)	0	0	0(0%)	0

Total		54743		677	240 (35.5%)	437		1214	101 (8.3%)	1113	189	93(49.21%)	96

^*a *^Candidate SNPs predicted by QualitySNP; ^*b *^candidate SNPs (score > = 50) predicted by autoSNP. confirmed: Candidate SNPs are considered as confirmed if they are present in dbSNP. Time (m): the minutes were used to run a program on each UniGene.

## Discussion

### Haplotype-based strategy for SNPs detection

We present here a SNP identification pipeline called QualitySNP that uses a haplotype-based strategy and reconstructs haplotypes at the start of the SNP identification process. This haplotype-based strategy makes full use of redundancy in sequences by clustering them, and in doing so not only reduces the influence of sequencing errors, but also removes poor quality sequences which otherwise would be identified as a haplotype with one single sequence. In the haplotype-based strategy, we eliminate SNPs that can be due to random and/or systematical sequencing errors (resulting from the sequencing strategy) or reverse transcriptase errors.

Once haplotypes have been defined and classified, it is possible to choose which SNPs will be used to diagnose the haplotype present in a genotype. Haplotype-based analysis of SNPs is more informative than analysis based on individual SNPs only, and is therefore more powerful in analyzing association with phenotypes [37].

### Haplotype reconstruction

About 96% of the clusters obtained from EST sequence data of potato were predicted to contain four or less haplotypes (Table 1), which shows that our haplotype definition based on potential SNPs works well. This agrees with the suggestion of Rafalski [37] and Russell *et al*. [38] that closely spaced SNPs will be sufficient to define haplotypes. Using the D-value to exclude clusters probably containing paralogs, only 3% of the remaining clusters still contained more than 4 haplotypes. This most likely results from incorrect haplotype reconstruction, which could be caused by several reasons. Firstly, some sequence errors may occur frequently due to systematic problems in the experimental procedures, and such repeated errors would be considered as valid alleles. Secondly, as EST sequences are usually much shorter than the corresponding mRNA, haplotypes at one end of the cluster sometimes cannot unambiguously be associated with haplotypes at the other end and will therefore be counted as separate haplotypes, raising the total number of haplotypes within one cluster. We checked ten clusters with more than 4 haplotypes and D-value less than 0.6, and found that for five clusters this was indeed the case. Thirdly, some paralogs may be highly similar, and may not be distinguishable from alleles. These paralogs may not be filtered out by filter 2, and account for the extra (false) haplotypes in a cluster.

### Paralogs identification

The identification of paralogs is an important problem in SNP detection, especially in large contigs, which are more likely to contain paralogs and random errors [10,16]. Most programs avoid the problem of large clusters by using a maximum cluster size of 20–50 for SNP discovery [10,16]. Our program is not limited by cluster size, and can handle clusters with an arbitrary number of members. SNPs in potentially interesting (highly expressed) genes are therefore still detected.

Paralogous sequences are generally less similar than allelic sequences. This property can be used to identify clusters that are likely to contain paralogs. POLYBAYES is a Bayesian method using the dissimilarity rate of paralogs and the polymorphism rate as input to calculate the probability that ESTs represent paralogs [9]. However, this may not be accurate when the polymorphism rate varies substantially between different genes. Our method detects clusters with paralogs by calculating the standard deviation (D) of the number of potential polymorphisms among haplotypes rather than the deviation from the mean polymorphism rate, and is therefore still able to reliably detect SNPs in highly variable allelic sequences. Discrimination of paralogs based on D-value is applicable if at least three haplotypes are detected in a cluster.

For our potato dataset, with the threshold D-value set to 0.6, 14% of the clusters with a high probability of containing paralogs were excluded from SNP detection. For the chicken dataset the D-value threshold set to 0.9, leaving 6% clusters with potentially paralogous sequences. The higher D-value threshold for the chicken dataset and the lower numbers of false positive and false negative clusters are most likely the result of the better quality of chicken sequence data compared to the potato data. In addition, the differences between chicken and potato may be partly accounted for by the fact that the potato genome is likely to contain more paralogous genes than the chicken genome, as gene duplication events in potato have occurred more frequently than in the chicken genome. Indeed, the paralog content of the chicken genome is relatively low even compared to the human genome [39].

The study with the human UniGene datasets (Table 2) demonstrates what the consequences are of different D-value threshold settings. When the D-value threshold was increased from 0.6 to 0.9, 53% (26) clusters confirmed with paralogs on the human genome with D-value threshold 0.6 were now wrongly designated allelic sequence clusters. Increasing the D-value will allow discovery of SNPs in additional clusters, but the percentage paralogous clusters of these additional clusters is also higher, which will decrease the reliability of the discovered SNPs. The most reliable set of SNP will therefore be produced at low D-values. Quality SNP enables the user to set D-value thresholds. This means that a user can decide to have the most reliable SNP dataset and use a low D-value threshold. However, if the user is interested in a gene that is represented in a cluster with a higher D-value, the D-value threshold can be increased, allowing this cluster to be investigated for SNPs by QualitySNP.

### Reliability of SNPs discovered by QualitySNP

Several steps in the QualitySNP pipeline are designed to improve the reliability of the SNP output of the program, while still being able to work with datasets from as many crops as possible (which means being able to produce highly reliable SNP identification even on datasets that do not have quality files). These steps include 1) the *mihap/mahap *calculations and settings and using the High Confidence Scores which effectively eliminates most of the SNPs identified in presumably low quality sequence regions (illustrated in Figure 5) and 2) haplotype reconstruction and using D-value thresholds for filtering out paralog-containing clusters (as illustrated by the data in Table 2). In QualitySNP, most of the settings can be adjusted according to the user's preference.

The reliability of SNPs produced by QualitySNP is illustrated by the fact that nearly all of the 52 potato SNPs (49) and an indel that we were able to evaluate by sequencing were indeed confirmed. In addition, our validation of the SNP output of QualitySNP using human EST and SNP data demonstrates that QualitySNP outperforms autoSNP, producing a higher number as well as more reliable SNPs than autoSNP. The percentage of SNPs confirmed by comparison to dbSNP may appear relatively low (35%). However, dbSNP is a public-domain archive for a broad collection of simple genetic polymorphisms (from NCBI), and although the number of SNPs in dbSNP increases everyday, it does not cover most of the SNPs present in the human genome. Therefore, it is likely that a number of true SNPs will not find a match in the dbSNPs, and therefore can not be confirmed.

### Retrieval system

QualitySNP includes a retrieval system that allows the user to extract additional useful information from the analysis. For instance, information about the nature of the SNPs (synonymous or non-synonymous) can be made part of the output. The SNP output can be modified by changing the reference genotype, and the D-value setting can be used to adjust the stringency with which paralogous clusters are detected and excluded. This may be very useful when focusing on a specific gene family where alleles of different paralogous sequences need to be identified. Statistics concerning the number of different types of SNPs and clusters can be included in the output. Searching parameters include the contig reference number, GenBank/EMBL/DDBJ accession number of ESTs, and UniGene ID; output options include SNP information, alignment information, EST function annotation information and ORF information of the contig. The SNP retrieval system based on the potato data is available at the website [32].

## Conclusion

In conclusion, QualitySNP works at least as well as, and in cases outperforms currently available methods, without the drawbacks of some of them, such as the necessity to provide a genomic sequence or sequence quality files. However, if quality files are available, this information can also be used by QualitySNP. By using a haplotype-based strategy, QualitySNP not only predicts reliable SNPs but also identifies haplotypes, and thus can be used in EST-based genotyping.

Another advantage of QualitySNP over other programs for SNP detection in nucleotide databases is the availability of a retrieval system that can output various kinds of data. Although QualitySNP can be used as a SNP detection tool with default settings, it can also be used for instance to examine specific clusters of genes, or to find nsSNPs in candidate genes.

## Authors' contributions

BV, JL and JT identified the need to develop the program, initiated the project, and designed its basic functionality. JT designed the algorithm and wrote the source code. All authors contributed to the overall design and feature requirements, and participated in the drafting of the manuscript and approved the final version.

## Supplementary Material

Additional file 1 QualitySNP. The source code of QualitySNP; The file is unpacked by using the command "gunzip QualitySNP.tar.gz", and then use "tar -xvf QualitySNP.tar" on a Unix/Linux computer. Click here for file

Additional file 2 testQualitySNP. A dataset for testing QualitySNP;The file is unpacked by using the command "gunzip testQualitySNPseq.tar.gz", and then use "tar -xvf testQualitySNPseq.tar" on a Unix/Linux computer. Click here for file

Additional file 3 QualitySNP manual. The manual of QualitySNP Click here for file
