# Supplementary material for: QualitySNP: a pipeline for detecting single nucleotide polymorphisms and insertions/deletions in EST data from diploid and polyploid species
Source: BMC Bioinformatics. 2006 Oct 9;7:438. doi: 10.1186/1471-2105-7-438 (PMC1618865; doi:10.1186/1471-2105-7-438)
Supplement: Additional file 1 — QualitySNP. The source code of QualitySNP; The file is unpacked by using the command "gunzip QualitySNP.tar.gz", and then use "tar -xvf QualitySNP.tar" on a Unix/Linux computer. [file 1471-2105-7-438-S1.gz › QualitySNPdir/website/help.html]

Help file for EST-SNP database

 

# EST-SNP database help page

## Search settings

- *SNP type* includes C/T, A/G, transition, A/T, A/C, C/G, T/G, transversion, indel and all of these.
  **required**
- *Advanced SNP type* includes
  non-synonymous SNP or synonymous SNP in the matching protein region or coding
  region, as well as SNP's in the UTR region. The results are based upon protein database
  searching
- The *reference species* is the species providing the EST resource. **required**
- *Search* database by
  contig ID, SNP ID, the gbid for the EST, or a unigene ID. All contig ID's with
  SNP can be chosen by selecting "all contigs with SNP", such as 24. SNP id
  is contigid\_snploci, such as "24\_49". Gbid is Genbank ID of one EST.
  Unigene ID is from the unigene db.
- The *D-value* is used to
  screen clusters/contigs with paralogs. The D-value shows the discrepancy
  of the SNP number among haplotypes (paralogs or orthologs groups). A higher D-value indicated
  that the cluster/contig is likely to contain paralogs. Most of D-value is in the interval [0,1]
  in our study.

## Output settings

- *include alignment info*
  shows alignment information of the contig and SNP location in the contig
  when searching by contig id

    For example:

- *include summary info*
  shows the statistical information about SNP of reference species
- *include SNP blocks*
  shows the blocks of SNP linked together in the contig.
  This works only when searching by contig id
- *include SNP pattern*
  shows all SNPs in the contig with same variation pattern.
  This works only when searching by contig id

- *include EST annotator*
  shows unigene, species and function information of all ESTs
  in the contig when searching by contig id
- *include SNP for microarray* shows SNP's that can be used for constructing probes for micro-array

- *all contigs with SNP*
  gives contig id, the number of EST, unigene id, and the number of true SNP's
  of all contigs with reliable SNP's
- *ORF info of all contigs with SNP* displays ORF information of all contigs with SNP
