# Supplementary material for: QualitySNP: a pipeline for detecting single nucleotide polymorphisms and insertions/deletions in EST data from diploid and polyploid species
Source: BMC Bioinformatics. 2006 Oct 9;7:438. doi: 10.1186/1471-2105-7-438 (PMC1618865; doi:10.1186/1471-2105-7-438)
Supplement: Additional file 1 — QualitySNP. The source code of QualitySNP; The file is unpacked by using the command "gunzip QualitySNP.tar.gz", and then use "tar -xvf QualitySNP.tar" on a Unix/Linux computer. [file 1471-2105-7-438-S1.gz › QualitySNPdir/QSNP_manual.pdf]

QualitySNP Pipeline Manual

Index

1. Introduction of QualitySNP
2. How to compile the programs
3. How to use the programs
4. How to use the results

1. Introduction to QualitySNP

QualitySNP is an efficient tool for SNP detection, storage and retrieval. It implements a new algorithm developed by us to reliably detect single nucleotide polymorphisms (SNPs) and insertions/deletions (indels) in expressed sequence tag (EST) data, both with and without quality files. The new algorithm uses a haplotype based strategy on potential SNPs, which predicts reliable SNPs, as well as reliable haplotypes.

The pipeline consists of six steps:

1) The first step performs EST assembling using **cross_match** for removing vectors and **CAP3** for sequence clustering.
2) The second step is the analysis of the alignment information to select clusters with at least 4 EST members; this is done by the Perl script "Getalignmentinfo". If sequences with quality information are available, another Perl script "Getalignmentinfoqual" is used instead of "Getalignmentinfo".
3) The third part performs SNP and haplotype detection, and distinguishes variations between or within genotypes. This is the core part of the pipeline, using the C program named "QualitySNP" that implements the algorithms for prediction haplotypes and SNPs. The helper programs "Getavailcontigseq" and "Getavailcontigqual" extract the sequences from the contigs and get the quality information of contigs.

In the case of sequences with quality information, the program "QualitySNPqual" should be used instead of "QualitySNP".

4) Step four is the non-synonymous SNP discovery using FASTY, from Pearson's FASTA package. A C program named "GetnonsySNPfasty" is used to analyze FASTY results, detect the ORFs and find non-synonymous SNPs.
5) The fifth step transfers the final results into a SNP database. It includes two C programs: "Getsnpindexcontig" (for sequences without quality information) or "Getsnpindexcontigqual" (for sequences with quality information) formats all information of contigs and the location and types of SNPs for insertion into the MySQL database; the second program "Transfersnpfasta" is used to convert SNP-containing probes for micro-array analysis into the format for the MySQL database.

   There are two SQL scripts to create the database and to load the data into the database. "dbcreater.sql" (for sequences without quality information) or "dbcreaterQ.sql" (for sequences with quality information) can create the database and tables that are used by the retrieval system, and "dataload.sql" puts the formatted data into database.
6) The final part is the retrieval system that is written in PHP. All PHP scripts and HTML pages are stored in the website's directory tree.

The QualitySNP pipeline itself is implemented in standard C-Shell script on a UNIX/Linux workstation. The Perl program is written in Perl5.8.0, the database uses MySQL3.23 (or better) and the retrieval system uses PHP4.3.1 (or better).

2. How to compile the programs

1) Download the packed file named `QualitySNP.tar.gz`

2) Unpack the tar file by the following command

```
% tar xzvf QualitySNP.tar.gz
```

or, if your tar command lacks the build-in unzip function, use

```
% zcat QualitySNP.tar.gz | tar xvf -
```

3) Compile the programs using the command

```
% make all
```

3. How to use the programs

There are two possible approaches to run these programs, the first way by running the programs manually one after the other, and the second way by running a C shell script name "QualitySNPpipeline" (see below).

I. The first (manual) way is as follows:

1) Make sure that the programs that you installed are in your search path. This can be done by adding the program-directory to your PATH variable. In the C-shell you can add the following to your "`.login`" file:

```
set path = ( my_program_dir $path )
```

and in the bash by adding the following to the "`.profile`" file:

```
export PATH=my_program_dir:$PATH
```

Of course, "`my_program_dir`" should be the path leading to your program directory (e.g. `/usr/local/bin/`).

2) Run CAP3 in a sequence file directory, containing the sequence file which includes all sequences in FASTA format. The CAP3 program can be obtained from http://genome.cs.mtu.edu/cap/cap3.html.

The commands are:

```
% formcon filename 500 4000
% cap3 filename -p similarity -o 100
```

The parameters in these commands are:

`filename` is the file with sequences in FASTA format, and

`similarity` is the similarity of overlap for CAP3.

For example, using a sequence file named `testseq`, and a similarity score of 95 like we used in our study, the commands would be:

```
% formcon testseq 500 4000
% cap3 testseq –p 95 –o 100
```

Please note: If sequences with quality information are used, the quality file with quality scores of all sequences should be formatted in FASTA format and should be named "`filename.qual`". For instance, for a sequence file named "`testseq`", the quality file should be named "`testseq.qual`".

3) Run Getalignmentinfo in the directory, by issuing the command:

```
% Getalignmentinfo filename.cap min-clustersize
```

where `filename` is the sequence file, and `min-clustersize` is the minimum cluster size. The default minimal cluster size is 4, you can set this according to your requirements. For example:

```
% Getalignmentinfo testseq.cap 4
```

Please note: if sequences with quality information are used, please run "Getalignmentinfoqual" instead of "Getalignmentinfo". The parameters of "Getalignmentinfoqual" are identical to those for "Getalignmentinfo".

4) Run QualitySNP in the same directory. The commands are:

```
% Getavailcontigseq filename.cap
% Getavailcontigqual filename.cap
% QualitySNP filename.cap min-allelesize lowqual5side
    similarity1 similarity2 lowqual3side weightlowqual
    min-confidencescore
```

The parameters used in these commands are:

`Min-allelesize` is the minimum size of alleles of each SNP (2 in our

study)

lowqual5side is the length of the low quality region at the 5' end of sequence (30 nucleotides in our study)

similarity1 is the similarity on one polymorphic site (0.75)

similarity2 is the similarity on all polymorphic sites (0.8)

lowqual3side is the low quality region of 3' side (0.2, 20% of the whole sequence in our study).

weightlowqual is the weight value of the low quality region (0.5)

min-confidencscore is the minimal confidence score（2）

For example:

```
% Getavailcontigseq testseq.cap
% Getavailcontigqual testseq.cap
% QualitySNP testseq.cap 2 30 0.75 0.8 0.2 0.5 2
```

Please note: if sequences with quality information are used, run "QualitySNPqual" instead of "QualitySNP". Besides parameters used by QualitySNP, one more parameter of min-SNPqualityscore is required by QualitySNPqual (the default value is 20 PHRED score). The usage of "QualitySNPqual" is the same as for "QualitySNP". For example:

```
% QualitySNPqual testseq.cap 2 30 0.75 0.8 0.2 0.5 2 20
```

5) Run FASTY and the analysis of non-synonymous SNP (this step is not necessary). FASTY can be obtained from http://fasta.bioch.virginia.edu/ as part of the FASTA package.

The command to run (the threaded version of) FASTY is:

```
% fasty34_t allavailcontigseqwithSNP Uniprot –b 6 –d 6 –Q > allavailcontigseqwithSNP.fasty
```

The command for the detection of non-synonymous SNPs is:

```
% GetnonsySNPfasty availcontigseq allavailcontigseqwithSNP allavailcontigseqwithSNP.fasty
```

Parameters used:

`Uniprot` is the Uniprot (or any other) protein database. This can be either the full path leading to a FASTA-formatted protein database, or a single letter to indicate the database, in case the FASTLIBS environment variable is used to specify databases in the FASTA suite.

The files "`availcontigseq`" and "`allavailcontigseqwithSNP`" are from the results of QualitySNP, File "availcontigseq" contains the consensus sequences of contigs with SNPs, as produced by CAP3. As these sequences are not curated, they may contain padding symbols ("*"), which may indicate either insertions and/or deletions in the ESTs, but in many cases these may be caused by sequencing errors. File "allavailcontigseqwithSNP" contains the consensus sequences of SNP-containing contigs which did not contain any insertions or deletions.

For example:

```
% fasty34_t allavailcontigseqwithSNP Uniprot –b 6 –d 6 –Q
> allavailcontigseqwithSNP.fasty
% GetnonsySNPfasty availcontigseq allavailcontigseqwithSNP
allavailcontigseqwithSNP.fasty
```

6) Format all information of SNPs and haplotypes, and transfer them to the database.

Commands for formatting data:

% Getsnpindexcontig filename

% Transfersnpfasta

Commands for moving results to one directory named "snpdb":

% mkdir snpdb

% mv estdata snpdb

% mv snpindexdata snpdb

% mv contigindexdata snpdb

% mv snpcontigdata snpdb

% mv allsnpmicro snpdb

Commands for creating databases and loading data:

If sequences without quality files, run the command:

```
% mysql –h ipaddress –u root –p < dbcreater.sql
```

If sequence with quality files, run the command:

```
% mysql –h ipaddress –u root –p < dbcreaterQ.sql
```

Data loading command for both cases:

```
% mysql –h ipaddress –u root –p < dataload.sql
```

Note: edit dataload.sql and change to the correct data directory

Parameters:

snpdb is the name of the directory for storing data

ipaddress is the IP address of computer to connect to carrying the mySQL database

For example:

```
% Getsnpindexcontig  testseq
% Tansfersnpfasta
```

Put all data to one directory "snpdb":

```
% mkdir snpdb
% mv estdata snpindexdata contigindexdata snpdb
% mv snpcontigdata allsnpmicro snpdb
```

Create the database and load the data into database for sequences without quality files:

```
% mysql -h 123.224.152.138 –u root –p < dbcreater.sql
% mysql -h 123.224.152.138 –u root –p < dataload.sql
```

Create the database and load the data into database for sequences with quality files:

```
% mysql -h 123.224.152.138 –u root –p < dbcreaterQ.sql
% mysql -h 123.224.152.138 –u root –p < dataload.sql
```

7) Set up your retrieval system

1. Start your apache server or other web server.
2. Put the website directory with PHP scripts and HTML pages in your server's "DocumentRoot" directory.
3. Edit reference species of the "search.php" page and put your species there. Edit "searchdb.php", put correct name of your database there

II. The second way of running the pipeline is MUCH easier: just run the C-shell script QualitySNPpipeline:

1. Edit QualitySNPpipeline program:

   Change `program_path1` to your alignment program CAP3 directory

   Change `program_path2` to your QualitySNP directory

   Change `ip address` to connect to your mySQL database

2. Run QualitySNPpipeline:

```
% path_to_QualitySNPdir/QualitySNPpipeline [-q] filename
similarityCAP3 min-clustersize min-allelesize lowqual5side
similarity1  similarity2  lowqual3side  weightlowqual
min-confidencescore
```

The parameters used are:

1. `filename` is the name of sequence file
2. `similarityCAP3` is the similarity of overlap for CAP3
3. `min-clustersize` of the minimum size of a cluster (4)
4. `min-allelesize` is the minimal size of alleles for each SNP (2)
5. `lowqual5side` is the size of the low quality region at the 5' side of the EST sequence (30 nucleotides in our study)
6. `similarity1` is the similarity on one polymorphic site (we used 0.75 in

our study)

7. similarity2 is the similarity on all polymorphism sites (0.8)
8. lowqual3side is the length of the low quality region at the 3' end (0.2; 20% of the whole sequence in our study)
9. weightlowqual is the weight value of the low quality region (0.5 in our study)
10. min-confidencscore is the minimum confidence score (2)

For example

```
% QualitySNPpipeline testseq 95 4 2 30 0.75 0.8 0.2 0.5 2
```

11. –q is the command line switch to instruct the pipeline to process sequences with quality information. The parameter MINQUAL in the pipeline is for the parameter min-SNPqualityscore of QualitySNPqual. The default value is 20 PHRED score, which can be changed by users as well (see line 58 of QualitySNPpipeline). The usage is as following:

```
% QualitySNPpipeline –q testseq 95 4 2 30 0.75 0.8 0.2 0.5 2
```

4. How to use the results

There programs will output a number of results files: some files contain information about SNPs and haplotypes, and others contain contig sequences and statistical information.

Important files for SNPs and haplotypes:

1. File "realsnpinfo" includes all SNPs in the clusters, excluding those from single haplotypes.
2. File "SNPquality" includes all relevant information for identifying reliable SNPs, such as confidence scores and allele haplotype scores, as well as quality score of SNPs if sequence quality available.
3. File "availcontigwithSNP" includes haplotype and SNP information for every contig, as well as statistical information of SNPs for all contigs

with SNPs.

Other information for SNPs haplotypes:

4. File “SNPinfo2” includes all potential SNPs for all clustered sequences.
5. File “haplotypes” includes all information for the haplotype definition.
6. File “SNPdiff” includes the D-values of the contigs, which are used for detecting paralogs.
7. File “allavailsnp” contains 25 nucleotides sense and reverse string surrounding the SNP, the SNP at the middle position (residue 13). These can be used for designing probes for inclusion on microarray chips.
8. File “SNPblocks” includes block information for every SNP in one cluster.
9. File “SNPpattern” includes pattern information for every SNP in one cluster.

Important files for contigs

1. File “contigstatisticinfo” includes the statistical information of cluster size and number of clusters. In this file, the first column is the number of ESTs in any cluster, the second column is the number of clusters of this particular size.
2. File “availcontigseq” contains the consensus sequences of contigs with SNPs, as produced by CAP3. As these sequences are not curated, they may contain padding symbols (“*”), which may indicate either insertions and/or deletions in the ESTs, but in many cases these may be caused by sequencing errors.
3. File “allavailcontigseqwithSNP” contains the consensus sequences of SNP-containing contigs which did not contain any insertions or deletions.
4. File “availcontiglist” shows a list of the contigs with sizes larger than the minimum cluster size, the number of ESTs in them, and length of

the longest EST therein.

5. File “availcontigseq” contains the sequence of the contigs whose size is larger than minimum cluster size.
6. File “availcontigqual” contains the quality scores of the contigs in file “availcontigseq”, as obtained from CAP3.
