# Supplementary figures and images for: QualitySNP: a pipeline for detecting single nucleotide polymorphisms and insertions/deletions in EST data from diploid and polyploid species
Source: BMC Bioinformatics. 2006 Oct 9;7:438. doi: 10.1186/1471-2105-7-438 (PMC1618865; doi:10.1186/1471-2105-7-438)

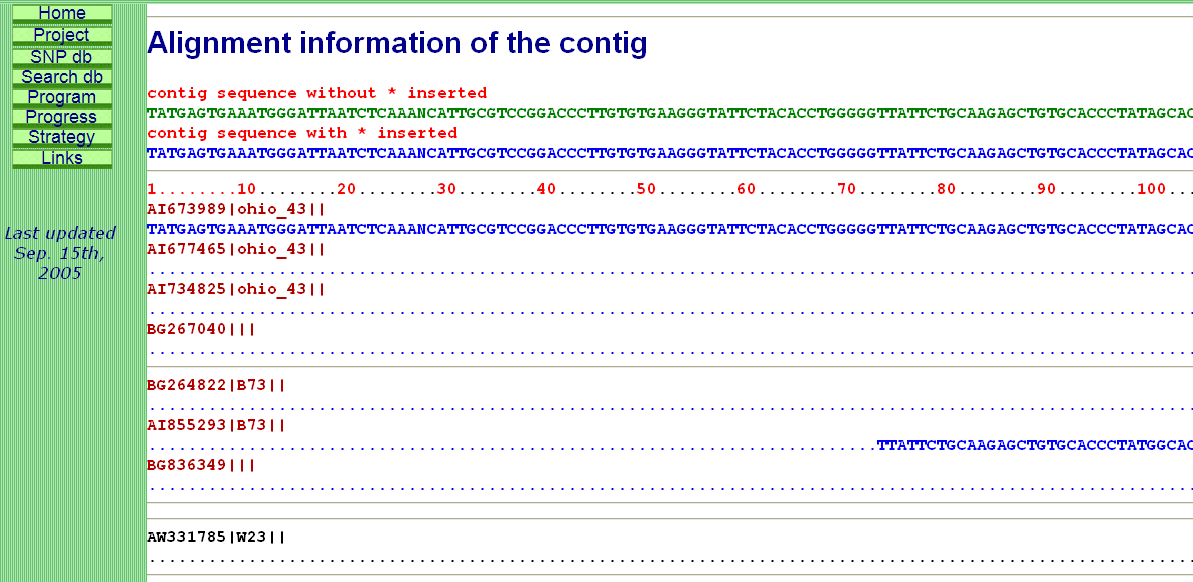

Supplement: Additional file 1 — QualitySNP. The source code of QualitySNP; The file is unpacked by using the command "gunzip QualitySNP.tar.gz", and then use "tar -xvf QualitySNP.tar" on a Unix/Linux computer. [file 1471-2105-7-438-S1.gz › QualitySNPdir/website/pictures/alignment.bmp]

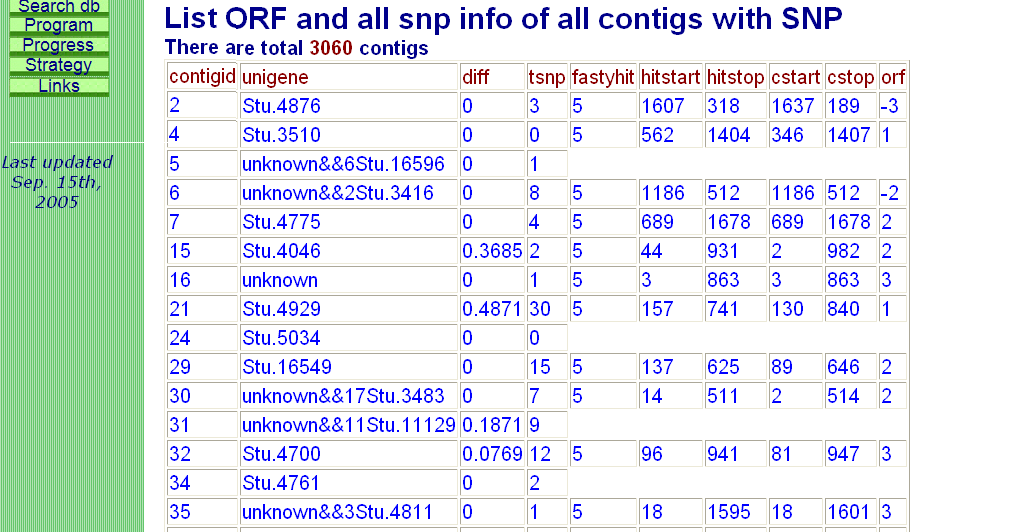

Supplement: Additional file 1 — QualitySNP. The source code of QualitySNP; The file is unpacked by using the command "gunzip QualitySNP.tar.gz", and then use "tar -xvf QualitySNP.tar" on a Unix/Linux computer. [file 1471-2105-7-438-S1.gz › QualitySNPdir/website/pictures/allcontigswithSNPs.bmp]

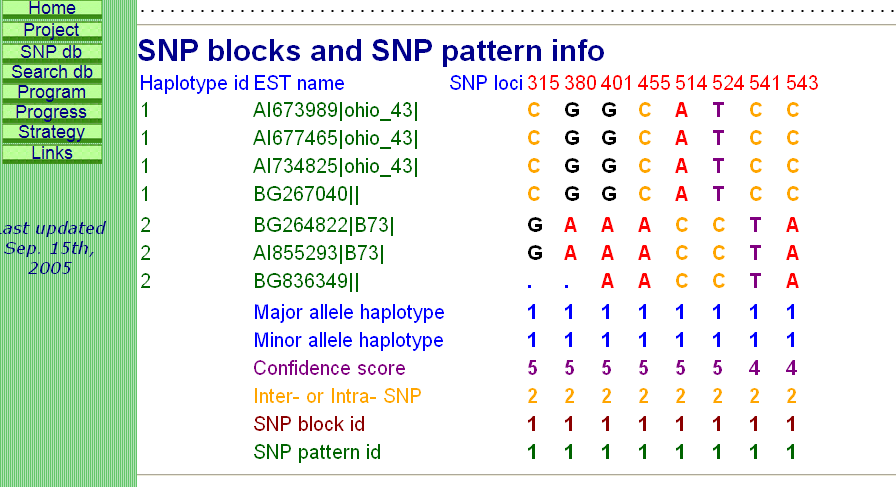

Supplement: Additional file 1 — QualitySNP. The source code of QualitySNP; The file is unpacked by using the command "gunzip QualitySNP.tar.gz", and then use "tar -xvf QualitySNP.tar" on a Unix/Linux computer. [file 1471-2105-7-438-S1.gz › QualitySNPdir/website/pictures/blockandpattern.bmp]

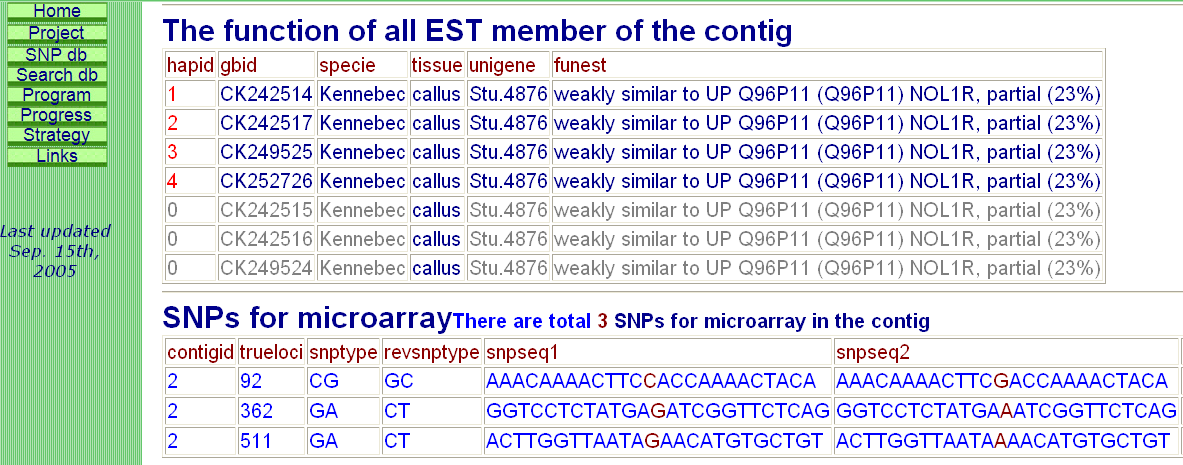

Supplement: Additional file 1 — QualitySNP. The source code of QualitySNP; The file is unpacked by using the command "gunzip QualitySNP.tar.gz", and then use "tar -xvf QualitySNP.tar" on a Unix/Linux computer. [file 1471-2105-7-438-S1.gz › QualitySNPdir/website/pictures/functionsnpmicroarry.bmp]
